# Insoluble and Soluble Dietary Fibers from Kiwifruit (*Actinidia deliciosa*) Modify Gut Microbiota to Alleviate High-Fat Diet and Streptozotocin-Induced TYPE 2 Diabetes in Rats

**DOI:** 10.3390/nu14163369

**Published:** 2022-08-17

**Authors:** Kunli Wang, Yuxiao Wang, Si Chen, Junlian Gu, Yuanying Ni

**Affiliations:** 1 School of Nursing and Rehabilitation, Cheeloo College of Medicine, Shandong University, Jinan 250012, China; 2 College of Food Science and Nutritional Engineering, China Agricultural University, Beijing 100083, China; 3 School of Food and Biological Engineering, Jiangsu University, Zhenjiang 212013, China

**Keywords:** kiwifruit, dietary fiber, type 2 diabetes mellitus, gut microbiota

## Abstract

This study aims to examine the anti-diabetic properties of insoluble and soluble dietary fibers from kiwifruit (KIDF and KSDF) in rats with type 2 diabetes mellitus (T2DM) resulting from a high-fat diet (HFD) and streptozotocin (STZ). Both KIDF and KSDF treatments for four weeks remarkably decreased body weight and increased satiety. In addition, the blood glucose level and circulatory lipopolysaccharide (LPS) content were decreased, while the insulin resistance, inflammatory status, and lipid profiles improved. These anti-diabetic effects might be related to the regulation of gut microbiota and increased SCFA content. The key microbial communities of KIDF and KSDF were different. Furthermore, the KIDF treatment increased the level of total SCFAs and isobutyric acid, while KSDF increased the levels of total SCFAs and butyric acid. The association between critical species and SCFA and between SCFA and biochemical parameters indicated that the mechanisms of KIDF and KSDF on T2DM might be different.

## 1. Introduction

Type 2 diabetes mellitus (T2DM) is one of the metabolic diseases resulting from defective insulin secretion and/or insulin action, and it makes up about 90% of all cases [1,2]. If the global prevalence of T2DM continued to rise, it is projected that an estimated 578 million people will have T2DM by 2030, and 700 million by 2045 [3].

According to recent studies, the composition and homeostasis of gut microbiota are associated with the development of diabetes [4]. Several molecular mechanisms have been proposed to elucidate this relationship. The high-fat diet (HFD) disrupts the balance of gut microbiota, which may impair intestinal function and increase serum endotoxin levels. This can result in chronic inflammation, leading to insulin resistance and T2DM [5,6,7]. Gut microbiota uses indigestible carbohydrates to ferment and produce short-chain fatty acids (SCFAs), such as acetic acid, propionic acid, and butyric acid. Such SCFAs strengthen the gut barrier function and enhance β-cell development and proliferation [8,9]. In addition, SCFAs can reduce appetite by increasing the levels of certain signaling proteins that are involved in modulating satiety to alleviate T2DM finally. Therefore, it is necessary to analyze the regulation of diabetes from the perspective of gut microbiota.

Diet has been established as an important target to prevent and treat T2DM and is one of the risk factors for T2DM [1]. Observational studies suggest that dietary fiber (DF) consumption was negatively correlated with the risk of T2DM [10]. The International Diabetes Federation recommends that sufficient consumption of DF should be part of a healthy diet or nutritional therapy to fight against T2DM [1]. DF is majorly composed of different carbohydrates that cannot be digested or absorbed in the small intestine but contribute to the maintenance of healthy gut microbiota. DF is categorized into two types, namely, soluble DF and insoluble DF, based on its water solubility.

Kiwifruit (*Actinidia deliciosa*) is a widely cultivated and economically important crop grown in many parts of China [11]. Previous studies have shown that kiwifruit is not only rich in vitamin C, polyphenols, and trace elements, but that it is also high in DF [12,13]. Our unpublished data suggest differences in monosaccharide composition between kiwifruit soluble dietary fiber (KSDF) and kiwifruit insoluble dietary fiber (KIDF) (Table S1). Several in vitro studies have shown that both KSDF and KIDF have good functional properties, such as glucose binding ability [14]. These results indicate that KSDF and KIDF have hypoglycemic potential. However, whether they can alleviate diabetes in vivo and regulate gut microbiota remains to be studied. This work aimed to evaluate the effects of KSDF and KIDF on T2DM based on the hypothesis that the alleviation was mediated by alterations in the gut microbiota as well as SCFAs generation. Hopefully, the current work provides certain experimental data on KSDF and KIDF as a kind of the functional food ingredient.

## 2. Materials and Methods

### 2.1. Materials

Hayward kiwifruits were supplied from Meixian Kiwifruit Experimental Station in Xian, China. Streptozotocin (STZ) was obtained from Sigma (St. Louis, MO, USA). Amyloglucosidase (300 AGU/g, AMG 300 L), protease (2.4 AU/g, Alcalase 2.4 L), and heat-stable α-amylase (300 KNU/g, Termamyl) were purchased from Novozymes Biotechnology Co., Ltd. (Tianjin, China).

### 2.2. Extraction of Insoluble and Soluble Dietary Fiber from Kiwifruit

The KIDF and KSDF were isolated, as described previously [14]. After pretreatment, the kiwifruits were ground and sieved (50 microns) to obtain kiwifruit powder (KP). About 0.2 mL of CaCl_2_ (1 M) and 400 mL of phosphate-buffered saline (PBS, pH 6) was added to 10.0 g KP, and mixed. Then, 500 µL Termamyl was added to the above mixture, followed by incubation for 1.5 h at 40 °C. Afterward, the solution was enzymatically digested using protease (500 µL, pH 7.5) for 1 h at 40 °C, followed by AMG (200 µL, pH 4.5) for 1 h at 40 °C. Following 15 min of centrifugation at 5000× *g*, all residues were extracted, rinsed twice using distilled water, and freeze-dried to obtain the KIDF. Meanwhile, the supernatants were collected, and a 4-fold volume of 95% ethanol was added and incubated for 2 h at ambient temperature to collect the residues. The residues were washed with 100% ethanol, followed by drying in a fume hood to obtain KSDF.

### 2.3. Animal Experiments

Adult male SD rats (200 ± 20 g) were obtained from Beijing Vitalstar Biotechnology Co., Ltd. (Beijing, China). In this work, all experimental protocols obtained approval from the Ethics Committee of Beijing Vitalstar Biotechnology Co., Ltd. (VST-SY-201912; Beijing, China). Rats were raised under standard laboratory conditions (temperature, 25 ± 2 °C; relative humidity RH, 45–65%; light-dark cycle, 12 h/12 h), and all animals had free access to food and water. Following acclimatized feeding for one week, six rats were randomized into a normal control group (NC) and raised with the normal chow diet (NCD, D12450J, supplemented with 10% kcal from fat, Research Diets Inc., New Brunswick, NJ, USA, Table S2) as well as intragastric administration of saline four weeks later. The remaining 30 rats were raised with HFD (D12492, supplemented with 60% kcal from fat, Research Diets Inc., New Brunswick, NJ, USA, Table S2) for four weeks. Later, the animals were given an intraperitoneal injection of STZ (30 mg/kg body weight BW). Fasting blood glucose (FBG) was determined at one week following STZ injection, and rats with high FBG (>11.1 mol/L) were chosen as T2DM model [15] and randomized as three groups: (1) diabetes model group (DM, *n* = 6), fed with the NCD as well as intragastrical administration of saline; (2) KIDF treatment group (KIDF, *n* = 6), fed with NCD along with intragastrical administration of 1000 mg/kg BW of KIDF for once a day; and (3) KSDF treatment group (KSDF, *n* = 6), fed with NCD along with intragastrical administration of 1000 mg/kg BW of KSDF once a day. All treatments were continued for four weeks, and food intake was measured twice a week, and BW for each rat was determined once weekly. At 9W7D, the 2 h postprandial blood samples were obtained, and serum contents of satiety hormones glucagon-like peptide 1 (GLP-1), and peptide YY (PYY) were determined using specific commercial kits (JYM0515Mo and JP40886, Jiyinmei Biotechnology Co., Ltd., Wuhan, China) following the manufacturer’s protocols. The rats were sacrificed following fasting for 16 h. Blood was sampled, followed by 15 min of centrifugation at 4000 rpm, 4 °C to isolate the serum [16]. The pancreatic samples were immediately excised and stored in 4% (*w/v*) paraformaldehyde. Feces and colon contents were collected and stored at −80 °C.

### 2.4. Biochemical Assays of Serum Samples

The levels of blood glucose (Glu), low-density lipoprotein (LDL), high-density lipoprotein (HDL), total cholesterol (TC), and triglyceride (TG) were determined using a fully automatic biochemical analyzer (Hitachi, Tokyo, Japan). In addition, insulin (Ins), interleukin-6 (IL-6), and tumor necrosis factor-α (TNF-α) contents were determined using ELISA kits (ab277390, ab222503 and ab208348, Abcam, Cambridge, MA, USA). Lipopolysaccharide (LPS) content was determined using commercial kits (JYM0588Mo, Jiyinmei Biotechnology Co., Ltd., Wuhan, China) following specific protocols. Homeostasis model assessment (HOMA)-insulin resistance (IR) were calculated according to the following formula:

HOMA-IR = GLU (mmol/L) × Ins (mIU/L)/22.5(1)

### 2.5. Histological Examination

Paraformaldehyde (4%) was used to fix the pancreas, followed by paraffin embedding. Pancreatic tissue sections were deparaffinized and stained with hematoxylin and eosin (H&E). The pathological changes in the lesion, together with the vicinity, were monitored using light microscopy.

### 2.6. Measurement of SCFAs in Colon Contents

About 100 mg of colon contents were resuspended in 1000 µL of 0.5% phosphoric acid. After centrifugation for 5 min at 10,000 rpm, the SCFAs in the supernatant was detected using GC–MS (Agilent 7890A/5975C, Agilent Technologies, Inc., Santa Clara, CA, USA) as described by Liu et al. [17].

### 2.7. Analysis of Gut Microbiota Based on 16S rRNA Gene Sequencing

The total bacterial genome DNA was extracted from fecal samples using the TIANamp Stool DNA Kit (DP328, TIAGEN, Beijing, China) [18]. Afterward, DNA quality and quantity were determined using the Nanodrop spectrophotometer (NC2000; Thermo Scientific, New York, NY, USA) and agarose gel electrophoresis, respectively. PCR amplification of V3–V4 regions of 16 S rRNA gene of gut microbiota was performed as described previously [19] using the primer sequences 5′-ACTCCTACGGGAGGCAGCAG-3′ (forward) and 5′-GGACTACHVGGGTWTCTAAT-3′ (reverse). For PCR amplification, a 20 µL reaction mixture was prepared (Table S3), and the PCR conditions were as follows: initial denaturation for 3 min under 95 °C, followed by 27 cycles of denaturation for 30 s under 95 °C, annealing for 30 s under 55 °C, and extension for 30 s under 72 °C, and final extension for 10 min under 72 °C. Then, 2% agarose gel was used to check all the PCR products, whereas the AxyPrep DNA Gel Extraction Kit (AP-GX-250G, Axygen Biosciences, Union City, CA, USA) was employed for purification. Finally, the QuantiFluor-ST software (Promega, Madison, WI, USA) was used to quantify the purified amplicons.

After the quality control analysis, the Illumina MiSeq sequences were acquired and imported into the Quantitative Insights Into Microbial Ecology software (QIIME, v1.9.1) to carry out subsequent analysis [20]. The raw 16S rRNA gene sequencing reads were demultiplexed, quality-filtered by fastp version 0.20.0 and merged by FLASH version 1.2.7 with the following criteria: (i) The 300 bp reads were truncated at any site receiving an average quality score of <20 over a 50 bp sliding window, and the truncated reads shorter than 50 bp and reads containing ambiguous characters were discarded; (ii) Only overlapping sequences longer than 10 bp were assembled according to their overlapped sequence. The maximum mismatch ratio of overlap region is 0.2. Reads that could not be assembled were discarded; (iii) Samples were distinguished according to the barcode and primers, and the sequence direction was adjusted, with exact barcode matching, and 2 nucleotide mismatch in primer matching. The operational taxonomic units (OTUs) were clustered using the UPARSE software (v 7.0.1090) based on 97% similarity threshold. At the same time, the Mothur software (v 1.30.2) was used for refraction and alpha diversity analyses, including Chao1, Ace, Simpson, and Shannon diversity indices. Principal coordinate analysis (PCoA) was utilized to assess the changes in experimental groups (β-diversity) using unweighted UniFrac analyses of R software. Mothur was used for the identification of critical communities for the isolation of diverse treatment groups using the LEfSe method, where Kruskal–Wallis was employed along with the Wilcoxon rank-sum test (*p* < 0.05). PICRUSt (v 1.1.0) was used for predicting the functional profiles of the gut microbiota [21]. Associations between SCFAs and critical gut microbial communities were analyzed using Spearman’s analysis (threshold, *p* < 0.05). Freely accessible Majorbio Cloud Platform (www.majorbio.com (accessed on 13 September 2021)) was employed for data analysis online.

### 2.8. Statistical Analysis

All values were presented as means ±SEM (standard error of the mean). SPSS 20.0 (IBM Corporation, New York, NY, USA) was used for statistical analyses. One-way analysis of variance (ANOVA) (Tukey’s test) was used for the analysis of experimental results. A difference of *p* < 0.05 indicated statistical significance.

## 3. Results

### 3.1. Results of Feeding Behavior and Satiety

**Figure 1:**
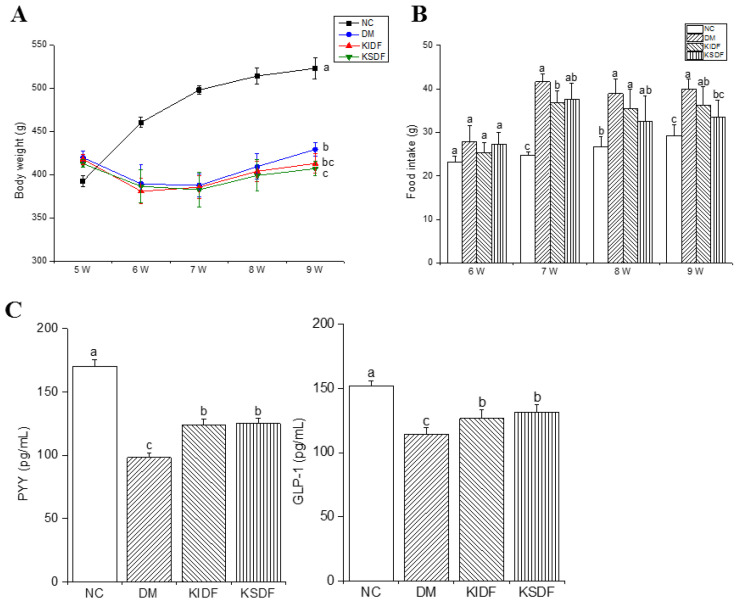
Effects of KIDF and KSDF on (**A**) body weight, (**B**) food intake, (**C**) PYY, and GLP-1 levels in the 2 h postprandial blood. Statistical significance was assessed using Tukey’s test for multiple comparisons. The different letters represent a significant difference (*p* < 0.05; the letters are marked in panel A for the 9th week). NC, normal control group; DM, diabetes model group; KIDF, kiwifruit insoluble dietary fiber treatment group; KSDF, kiwifruit soluble dietary fiber treatment group.

The rats were injected with STZ at week 5 of modeling induction and fed with NCD from week 6 to week 9. The results showed that the rats lost weight sharply during the modeling process, and KSDF significantly reduced the weight of diabetic rats after the 4-week treatment (Figure 1A). In addition, from week 7, the food intake of the fiber-fed rats was reduced compared with those in the DM group (Figure 1B). This suggests that both KIDF and KSDF could enhance satiety. Similarly, KIDF and KSDF elevated PYY and GLP-1 contents after treatment compared with the DM group (Figure 1C).

### 3.2. Serum Biochemical Parameters

Table 1 presents Glu, insulin, and serum indices after four weeks of KIDF and KSDF supplementation. Compared to the DM group, both KIDF and KSDF significantly reduced TG, LPS, TNF-α, and IL-6 concentration. In addition, only KSDF treatment remarkably decreased Glu, TC, and LDL contents. The insulin levels in the KIDF and KSDF groups were improved relative to DM and NC groups but with no significant difference. However, HOMA-IR was decreased after both KIDF and KSDF, indicating that insulin resistance is reduced. There were no observed differences in HDL and LPS among the three groups.

### 3.3. Histological Changes in the Pancreas

**Figure 2:**
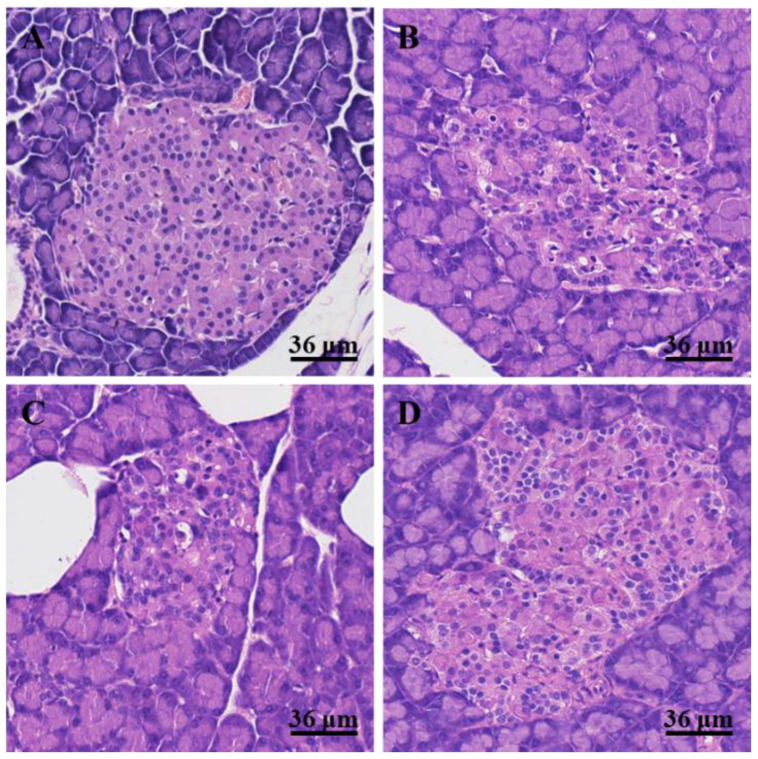
The H&E staining of the pancreas (HE 40×) in four groups. (**A**) NC, normal control group; (**B**) DM, diabetes model group; (**C**) KIDF, kiwifruit insoluble dietary fiber treatment group; (**D**) KSDF, kiwifruit soluble dietary fiber treatment group.

The pancreatic sections stained with H&E are shown in Figure 2. No inflammatory cell infiltration, degeneration of acinar cells, and edema were observed in the pancreatic islets of rats in the NC group, and no steatosis was observed in acinar cells (Figure 2A). However, degeneration of acinar cells and inflammatory cell infiltration were observed in the pancreatic islets of HFD/STZ-induced T2DM rats (Figure 2B). After KIDF treatment, the morphologies of pancreatic islets were still irregular with a degree of inflammatory cell infiltration. The pancreatic islet cells were denatured with mild edema (Figure 2C). The KSDF treatment notably improved the morphologies of pancreatic islets (Figure 2D). A small amount of infiltration of inflammatory cells was observed, and the pancreatic islet cells were slightly eosinophilic.

### 3.4. SCFA Concentration in the Gut

The major metabolic products of dietary carbohydrates are SCFAs. As shown in Table 2, HFD/STZ treatment reduced the SCFAs content compared to the NC group. KIDF and KSDF had different effects on SCFAs compared to the DM group: KSDF treatment significantly increased butyric acid and total SCFA levels, whereas KIDF treatment markedly elevated isobutyric acid level.

### 3.5. Composition of Gut Microbiota

After removing unqualified sequences, 24 samples from four groups generated 1,313,859 sequences altogether, with 57,663 sequences for each NC sample, 48,374 for each DM sample, 55,504 for each KSDF sample and 57,434 for each KIDF sample on average, with the mean sequence length of 401–440 base pairs (Figure S1A). Among them, those that qualified (>0.001%) were classified as 729 bacterial OTUs (Figure S1B). As shown in Figure S1B, there were 119, 14, 13, and 15 unique OTUs found in the NC, DM, KIDF, and KSDF groups, respectively.

The alpha diversity (Chao1, ACE, Simpson, and Shannon index, Figure S2) was used to analyze the overall structure of the gut microbiota. The Shannon index was reduced in the DM group compared to the NC group, while the Simpson index was increased. After KIDF and KSDF supplementation, both Simpson and Shannon index were close to those of NC rats. No significant alteration was observed in the ACE and Chao 1 index among the four groups. The beta diversity metric (Principal Coordinate Analysis, PCoA) using unsupervised multivariate statistical methods was used to analyze the gut microbial heterogeneities of different treatments (Figure S3). PCoA plots demonstrated that gut microbiota in the NC group was significantly different from those of the other three groups, along with the first principal component (PC1). Additionally, the microbial community structure in the DM group slightly shifted toward the second principal component (PC2) with KIDF and KSDF treatment. Taken together, these results revealed that KIDF and KSDF supplements affected the gut microbiota profiles of T2DM rats.

### 3.6. KIDF and KSDF Modulate Gut Microbiota Imbalance of HFD/STZ Induced T2DM Rats

**Figure 3:**
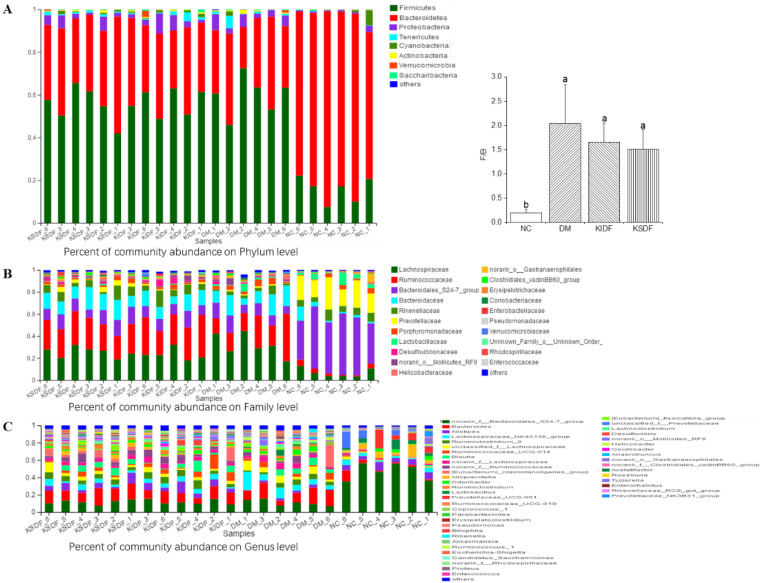
The relative abundance of gut microbiota at the phylum, family, and genus levels. Effects of NC, DM, KIDF and KSDF on the percentage of community abundance on (**A**) phylum, (**B**) family, and (**C**) genus levels. The diverse letters represent a significant difference (*p* < 0.05). F/B, Firmicutes/Bacteroidetes.

The different relative abundances for diverse microorganisms across various groups were examined. Firmicutes and Bacteroidetes, the two most dominant phyla, were predominant in all the 24 samples (Figure 3A). The ratio of Firmicutes to Bacteroidetes (F/B) was remarkably elevated in the DM group in comparison to the NC group. The F/B ratio was decreased in both KIDF and KSDF rats compared to DM rats, even though the differences were not significant. At the family level (Figure 3B), rats under both KIDF and KSDF treatment had a higher relative abundance of *Lachnospiraceae*, *Ruminococcaceae*, *Bacteroidales_S24–7_group*, *Bacteroidaceae*, *Prevotellaceae*, *Lactobacillaceae*, and *Desulfovibrionaceae* compared with those of NC rats. At the genus level (Figure 3C), DM rats had remarkably elevated relative abundances for *Bilophila* and *Lachnoclostridium* compared to NC rats, while KSDF treatment decreased the relative abundance of the above bacteria. Furthermore, lower levels of *Alloprevotella* and *Alistipes* were observed in the DM group than in the NC group, while KSDF treatment increased the relative abundance of the above bacteria, and KIDF only increased the relative abundance of *Alistipes*.

Previous studies have shown that *Lachnospiraceae* and *Ruminococcaceae* can decompose various refractory polysaccharides and are beneficial for intestinal barrier function [22], which are two families with the highest abundance in phylum *Firmicutes*. At the family level (Figure 3B), the KIDF and KSDF groups showed an increased relative abundance of *Ruminococcaceae* (29.86% and 26.28%, respectively) than the DM groups (22.98%), but the differences were not significant. Figure 3C showed that the relative abundance of *Ruminococcaceae_UCG-014*, *Acetatifactor*, *Tyzzerella*, *norank_f__Ruminococcaceae*, *Lachnospiraceae_NK4A136_group*, *Oscillibacter*, and *unclassified_f__Lachnospiraceae* were especially improved after KIDF or KSDF treatment.

**Figure 4:**
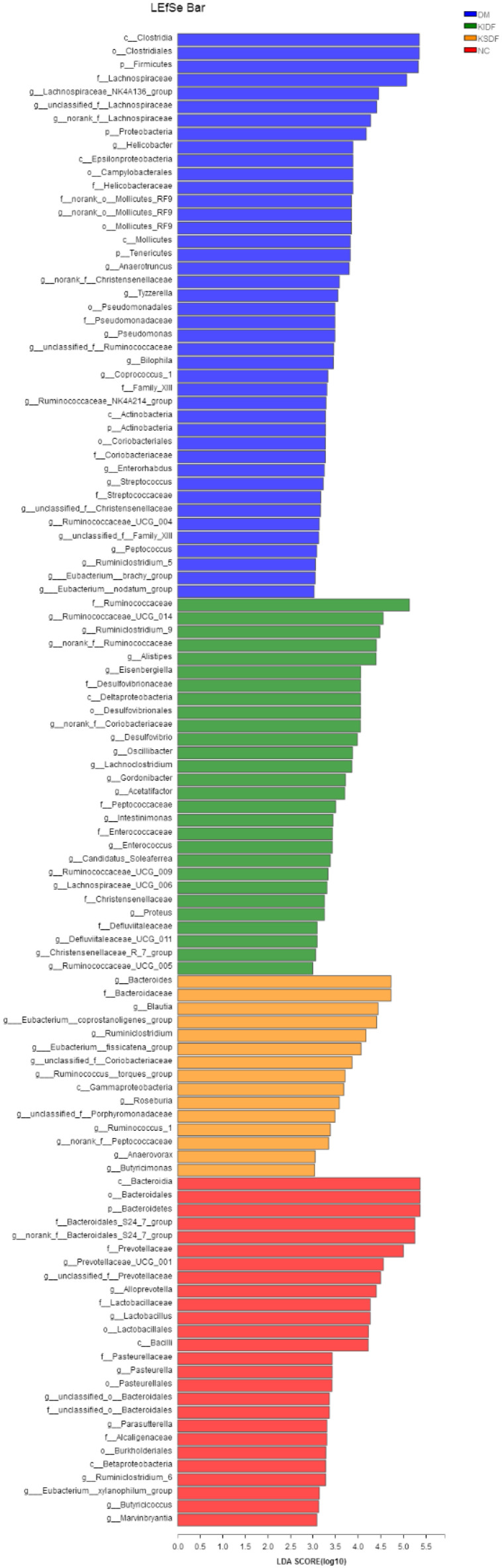
Linear discriminant analysis (LDA) score for taxa differing between the four groups. An LDA score greater than 2 indicated a higher relative abundance in the corresponding group than in the other three groups. Blue bars represent taxa that are significantly increased in the DM group. Green bars represent taxa that are significantly increased in the KIDF group. Orange bars represent taxa that are significantly increased in the KSDF group. Red bars represent taxa that are significantly increased in the NC group.

The key communities among the four groups were determined by LEfSe by Kruskal–Wallis and the Wilcoxon rank-sum test (*p* < 0.05) (Figure S4) and the linear discriminant analysis (LDA) (Figure 4). KSDF revealed that nine communities were selectively enriched, which were *Butyricimonas*, *Blautia*, *Roseburia*, *Ruminococcus_1*, *Ruminiclostridium* et al. KIDF suggested the potent effects on 18 communities that belonged to the *Lachnospiraceae* and *Ruminococcaceae* families. T2DM induced bacteria, including *Family_XIII* family, as well as including *Bilophila*, *Helicobacter*, *Streptococcus* and *Enterorhabdus*.

### 3.7. Correlations of Critical Gut Microbial Communities with the Biochemical Parameters

**Figure 5:**
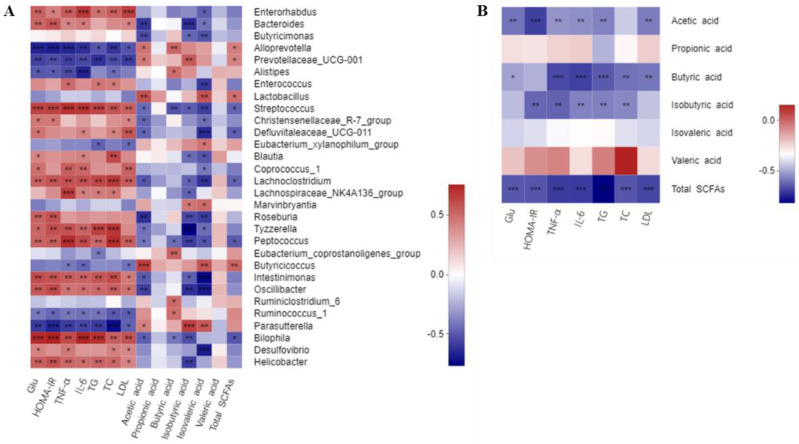
(**A**) Correlation between biochemical parameters and key communities of gut microbiota obtained from LEfSe results. (**B**) Correlation between biochemical parameters and SCFAs. Spearman’s correlation coefficients and *p* values for the correlations are calculated (* *p* < 0.05, ** *p* ≤ 0.01, *** *p* ≤ 0.001). A positive correlation is shown in red, and a negative correlation is shown in blue.

To investigate the possible correlation of changes in the gut microbiota with the host phenotypes, and also to determine whether KIDF and KSDF generated SCFAs together with heterogeneities in the gut microbiota, the associations between biochemical parameters and critical communities were examined based on LEfSe results (Figure 5A, Table S5). *Alloprevotella*, *Prevotellaceae_UCG-001*, *Lactobacillus*, *Butyricicoccus*, and *Parasutterella* showed a positive correlation with acetic acid, and the Spearman’s correlations were 0.47, 0.45, 0.59, 0.65 and 0.43, respectively. *Alloprevotella*, *Alistipes*, *Eubacterium_coprostanoligenes_group*, *Ruminiclostridium_6* and *Ruminococcus_1* showed a positive correlation with butyric acid, and the Spearman’s correlations were 0.52, 0.49, 0.53, 0.50 and 0.49, respectively. *Marvinbryantia* and *Parasutterella* showed a positive correlation with both isobutyric acid and isovaleric acid. *Alloprevotella*, *Prevotellaceae_UCG-001*, *Lactobacillus*, and *Butyricicoccus* showed a positive correlation with total SCFAs. All these microbiotas showed a negative correlation with Glu, HOMA-IR, TG, TC, and inflammation levels. On the contrary, *Enterorhabdus*, *Bacteroides*, *Streptococcus*, *Defluviitaleaceae_UCG-011*, *Peptococcus*, *Oscillibacter*, *Bilophila*, and *Helicobacter* showed a significant negative correlation with SCFA content or a positive correlation with levels of Glu, HOMA-IR, TG, TC, and inflammation.

### 3.8. Correlations between the Contents of SCFAs and Biochemical Parameters

A strong negative correlation (*p* < 0.0001) was found between butyric acid and TNF-α, IL-6, and TG (Figure 5B, Table S5), and the correlation between butyric acid and other biochemical parameters was negative. Furthermore, acetic acid, isobutyric acid, and total SCFAs had negative correlations with these biochemical parameters.

## 4. Discussion

After four weeks of dietary interventions, both KIDF and KSDF resulted in decreased levels of blood glucose, ameliorated insulin resistance, and improved tissue morphology of the pancreas. We note that KSDF improved the morphologies of pancreatic islets, while Glu levels and other biochemical parameters were only partially improved. This might be caused by the inter-individual variability and sample selection. This suggests that we could use electron microscopy to observe the ultrastructure of pancreatic β-cells and more accurately determine the extent of islet destruction. Additionally, we observed decreased serum TC, TG, and LDL-C levels when treated with both KIDF and KSDF. We also observed that SCFAs had negative correlations with TC, TG, and LDL-C levels (Figure 5B). The results were consistent with a previous study [1], which showed that SCFAs fermented by DF may be the cause of improved lipid metabolism. Moreover, the results of food intake indicated that supplementing rat feed with KIDF and KSDF promoted satiety, and the contents of PYY and GLP-1 also proved this. Our results are consistent with the results of Tan et al. [23], demonstrating that 2% of combined soluble fiber reduced food intake by promoting satiety. In addition to the effect of hormones (PYY and GLP-1), the decrease in food intake was associated with DF properties. DF exhibits good water absorption, expansibility, and can increase the viscosity of chyme. Therefore, it increases satiety to some extent and slows stomach emptying. A prolonged caloric surplus is the primary cause of obesity [24,25]. Therefore, KIDF and KSDF also have the potential to reduce obesity. However, in the present study, KSDF and KIDF intervention seemed to delay weight gain in rats. Therefore, whether DFs affect the weight gain of rats still needs to be further explored.

Systemic inflammation plays an important role in the occurrence of metabolic syndrome, suggested by growing evidence. Impaired gut barrier function induced by HFD can increase endotoxin levels of LPS in circulating systems, and LPS upregulates the expression of inflammatory cytokines [26]. In general, insulin resistance is directly or indirectly caused by increased inflammation [6]. Systemic inflammation damages the pancreatic β cells, disrupts insulin action and mediates glucose intolerance [5]. In this study, both KIDF and KSDF supplementation decreased serum LPS levels and inflammation induced by HFD. It is worth mentioning that KSDF had a better effect than KIDF in suppressing inflammation.

With improvements in blood glucose and lipid levels, we observed altered gut microbiota composition and differences in key microbial communities in the different treatment groups. To the best of our knowledge, this is the first time sequencing analysis of microbial 16S rRNA has been used to study the effects of kiwifruit dietary fibers on gut microbiota in T2DM rats in vivo. The results indicated that the HFD/STZ-induced T2DM rats have significantly separated clusters of the gut microbiota and significantly decreased alpha diversity compared to the NC group rats, while KIDF and KSDF increased the intestinal microbial diversity of the diabetic rats. Compared with nondiabetic rats, a higher F/B ratio and increased abundance of *Proteobacteria* were observed in DM group rats, which is consistent with the previous reports [27,28]. These major phyla may be associated with inflammation, insulin resistance, and elevated blood glucose level [4]. Although the differences did not reach a significant level, the F/B ratio was decreased in both KIDF and KSDF rats compared to DM rats, which suggests that both KIDF and KSDF might exert anti-diabetic effects via modulation of gut microbiota. Additionally, at the genus level, KIDF and KSDF interventions were shown to remodel the gut microbiota imbalance caused by T2DM. In particular, KIDF and KSDF interventions increased the relative abundance of SCFA-producing beneficial bacteria such as *Alloprevotella*.

Although the species have a similar shift when supplemented with KIDF and KSDF, the key communities are different for these two treatments. Particularly, *Butyricimonas*, *Blautia*, *Roseburia*, *Ruminococcus_1*, and *Ruminiclostridium* have been reported to degrade complex polysaccharides into SCFAs [4,29], especially, acetic acid and butyric acid. Among the four groups, only KSDF showed a selective increase in these species. KIDF showed significant selective enrichment of *Oscillibacter*, *Alistipes*, and *Desulfovibrio*, which were found in the intestines of mice fed with DF or complex polysaccharides [4,30]. Furthermore, *Helicobacter*, *Streptococcus*, *Enterorhabdus*, and *Bilophila* were key communities in the HFD group. The increase of *Enterorhabdus* and *Streptococcus* is associated with cancer, intestinal injury, and other diseases [31,32], while the increase in *Helicobacter* may increase the probability of gastric ulcer and gastric cancer [33]. Our results also found that all these species had a positive association with T2DM characteristics, such as a high level of blood glucose, insulin resistance, inflammation, and hyperlipidemia (Figure 5A). However, Zhong et al. [34] found that supplementation of whole-grain barley and barley malt increased the relative abundance of *Bilophila*, which is inconsistent with our experimental results.

SCFAs are produced by gut microbial fermentation of indigestible polysaccharides [35], which can improve the symptoms of T2DM and obesity by affecting the lipid and energy metabolism [36,37]. Two SCFA receptors (FFA2 and FFA3) are metabolic sensors of the pancreatic β cells. Moreover, longer SCFAs (4–6 carbons) and SCFAs with branched alkyl groups (isobutyrate) can activate FFA3 better than FFA2 [38]. Our study demonstrated that the levels of butyric acid, isobutyric acid, and total SCFAs after treatment with kiwifruit DF were higher than in the DM group, and they were significantly negatively correlated with Glu, Homa-IR, and lipid levels. Therefore, the hypolipidemic and alleviation effect of KSDF and KIDF on T2DM is associated with the activation of FFA3 by SCFAs. We also found that the gut microbiota with a significant positive correlation with butyric acid content in this experiment were the key communities in the KIDF and KSDF treatment groups and the NC group. However, in the two treatment groups, only the level of butyric acid was significantly higher in the KSDF group than that of diabetic rats. Butyrate increases insulin sensitivity and reduces glucagon production in the pancreas and stimulates glucose uptake in the muscle and adipose tissue [29], so butyrate supplementation is important to treatment or prevention of diabetes [39]. Moreover, KSDF treatment had higher contents of total SCFAs than KIDF. This is consistent with the previous study that soluble DF is more likely to be fermented in the gut to produce SCFAs than insoluble DF [40]. It should be noted that the isobutyric acid levels after KIDF treatment were higher than in the DM and KSDF groups. This may be the product of KIDF fermentation by intestinal microbiota. Another explanation is that insoluble DF may affect protein absorption [41]. Therefore, KIDF may reduce the absorption and utilization of protein in food, which was fermented by gut microbiota to produce isobutyric acid. Further evidence is needed to confirm the source of isobutyric acid.

The results of environmental factor correlation analysis showed that specific gut microbiota were negatively correlated with biochemical correlates and the inflammatory factor of T2DM, and correlated with the concentration of SCFAs. Therefore, combined with the above analysis of the effects of SCFAs, our findings suggest that KSDF and KIDF treatment had effect on T2DM indicators by changing gut microbiota and its metabolite. Monosaccharide composition and glycosidic bond type are important characteristics of DF. According to our previous experimental data, KSDF has a high proportion of galactose and galacturonic acid, while KIDF has a high proportion of xylose and glucose. However, which monosaccharide and glycosidic bonds in KSDF and KIDF affect gut microbiota remains to be further explored.

## 5. Conclusions

In conclusion, the results of biochemical parameters demonstrate the improved effects of KSDF and KIDF treatment against T2DM, and KSDF has a better anti-diabetic effect than KIDF. The mechanism of this effect may be associated with changes in the gut microbiota. During the treatment of HFD/STZ-induced T2DM in rats, KSDF and KIDF showed a difference in the enrichment of key gut microbial communities and SCFAs contents. This study showed that KSDF and KIDF might improve T2DM via different mechanisms, but eventually, both of them mediate their beneficial effects on the host.

## Figures and Tables

**Table 1:** The effects of KIDF and KSDF on blood biochemical parameters.

Group	NC	DM	KIDF	KSDF
Glu (mmol/L)	5.25 ± 0.26 c	27.25 ± 4.23 a	24.32 ± 4.08 ab	21.15 ± 3.02 b
Ins (μIU/mL)	22.01 ± 2.70 b	39.69 ± 3.21 a	36.71 ± 4.23 a	36.91± 4.16 a
HOMA-IR	5.15 ± 0.78 c	48.29 ± 9.66 a	39.63 ± 8.28 ab	34.56 ± 5.46 b
TG (mmol/L)	0.47 ± 0.03 c	1.81 ± 0.16 a	1.12 ± 0.43 b	0.82 ± 0.38 bc
TC (mmol/L)	1.66 ± 0.15 c	5.64 ± 0.8 a	4.59 ± 0.52 ab	3.9 ± 0.86 b
LDL (mmol/L)	0.34 ± 0.14 c	3.08 ± 0.7 a	2.79 ± 0.57 ab	1.86 ± 0.68 b
HDL (mmol/L)	1.34 ± 0.23	1.22 ± 0.25	1.26 ± 0.27	1.25 ± 0.33
LPS (ng/mL)	90.53 ± 2.61 d	187.50 ± 7.74 a	175.05± 6.53 b	161.74 ± 6.62 c
TNF-α (pg/mL)	14.78 ± 0.63 c	44.99 ± 1.48 a	35.61 ± 3.40 b	32.97 ± 1.82 b
IL-6 (pg/mL)	96.49 ± 1.77 c	290.37 ± 2.91 a	247.66 ± 4.28 b	239.84 ± 7.89 b

NC: normal control group; DM: diabetes model group; KIDF: kiwifruit insoluble dietary fiber treatment group; KSDF: kiwifruit soluble dietary fiber treatment group. Data are presented as mean ± SD. The different letters in the same row represent the significant difference, *p* < 0.05.

**Table 2:** Effects of KIDF or KSDF on SCFAs produced in gut.

Concentration (μg/mg)	NC	DM	KIDF	KSDF
Acetic acid	1.56 ± 0.08 a	1.17 ± 0.17 b	1.21 ± 0.20 b	1.26 ± 0.16 b
Propionic acid	1.50 ± 0.24	1.32 ± 0.22	1.39 ± 0.19	1.38 ± 0.23
Butyric acid	0.90 ± 0.06 a	0.45 ± 0.14 b	0.63 ± 0.10 b	1.05 ± 0.12 a
Isobutyric acid	0.09 ± 0.01 a	0.06 ± 0.01 c	0.08 ± 0.01 b	0.06 ± 0.01 c
Isovaleric acid	0.09 ± 0.01 a	0.06 ± 0.01 b	0.05 ± 0.01 c	0.06 ± 0.00 c
Valeric acid	0.04 ± 0.01	0.03 ± 0.01	0.04 ± 0.00	0.04 ± 0.00
Total SCFAs	4.18 ± 0.33 a	3.11 ± 0.38 c	3.39 ± 0.25 bc	3.84 ± 0.22 ab

NC: normal control group; DM: diabetes model group; KIDF: kiwifruit insoluble dietary fiber treatment group; KSDF: kiwifruit soluble dietary fiber treatment group. Data are presented as mean ± SD. The different letters in the same row represent the significant difference, *p* < 0.05.

## Data Availability

All data that support the findings of this study are available from the corresponding author on reasonable request.

## Supplementary Materials

The following supporting information can be downloaded at: https://www.mdpi.com/article/10.3390/nu14163369/s1, Figure S1: DNA raw data and Comparison of the OTUs in the four groups.; Figure S2: Effects of KIDF and KSDF on the community richness and diversity of gut microbiota in mice; Figure S3: Effects on the gut microbiota structure by beta diversity metric using unsupervised multivariate statistical methods with principal coordinate analysis (PCoA); Figure S4: he significantly different species were determined based on the LEfSe method using the nonparametric factorial Kruskal–Wallis rank sum test at a significance level of 0.05.; Table S1: Monosaccharide composition of KSDF and KIDF; Table S2: Ingredients and energy densities of experimental diets; Table S3: Component of the PCR reaction system; Table S4: Correlation of key communities of gut microbiota and biochemical parameters in NC, DM, KIDF and KSDF groups; Table S5: Correlation of SCFAs and biochemical parameters in NC, DM, KIDF and KSDF groups.
