# Supplementary material for: Insoluble and Soluble Dietary Fibers from Kiwifruit (Actinidia deliciosa) Modify Gut Microbiota to Alleviate High-Fat Diet and Streptozotocin-Induced TYPE 2 Diabetes in Rats
Source: Nutrients. 2022 Aug 17;14(16):3369. doi: 10.3390/nu14163369 (PMC9414595; doi:10.3390/nu14163369)
Supplement: Supplementary file 1 [file nutrients-14-03369-s001.zip › nutrients-1859739-supplementary.pdf]

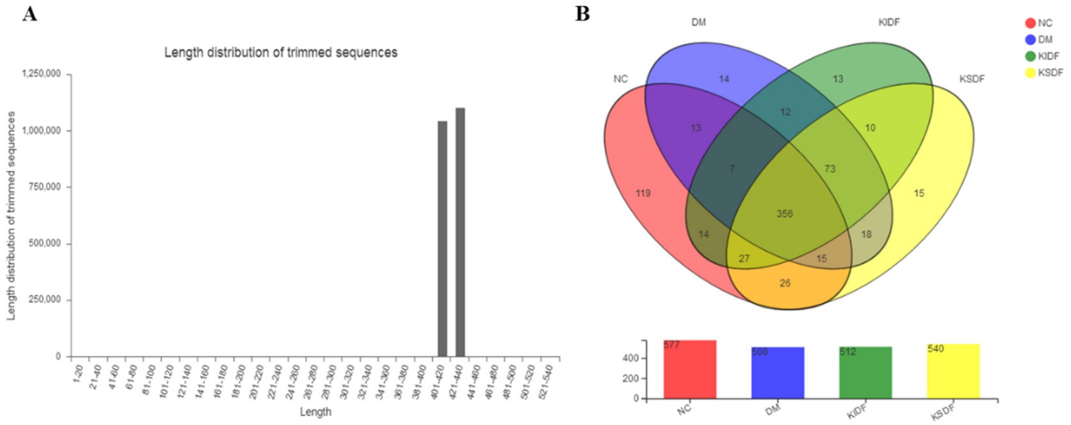

Figure S1. DNA raw data and Comparison of the OTUs in the four groups. The number of observed OTUs sharing >97% nucleotide sequence identity. (**A**) DNA sequencing number and length; (**B**) A Venn diagram was generated to describe the common and unique OTUs among the normal control group (NC, n = 6), diabetes model group (DM, n = 6), kiwifruit insoluble dietary fiber treatment group (KIDF, n=6) and kiwifruit soluble dietary fiber treatment group (KSDF, n = 6).

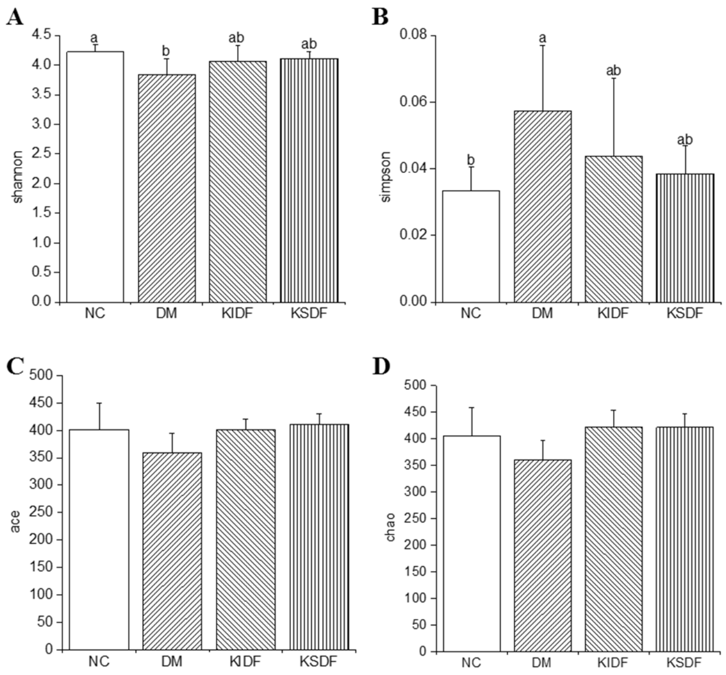

Figure S2. Effects of KIDF and KSDF on the community richness and diversity of gut microbiota in mice: (**A**) the shannon index; (**B**) the simpson index; (**C**) the ace index; (**D**) the chao index. Normal control group, NC; diabetes model group, DM; kiwifruit insoluble dietary fiber treatment group, KIDF; kiwifruit soluble dietary fiber treatment group, KSDF. The diverse letters represent significant difference ($p < 0.05$).

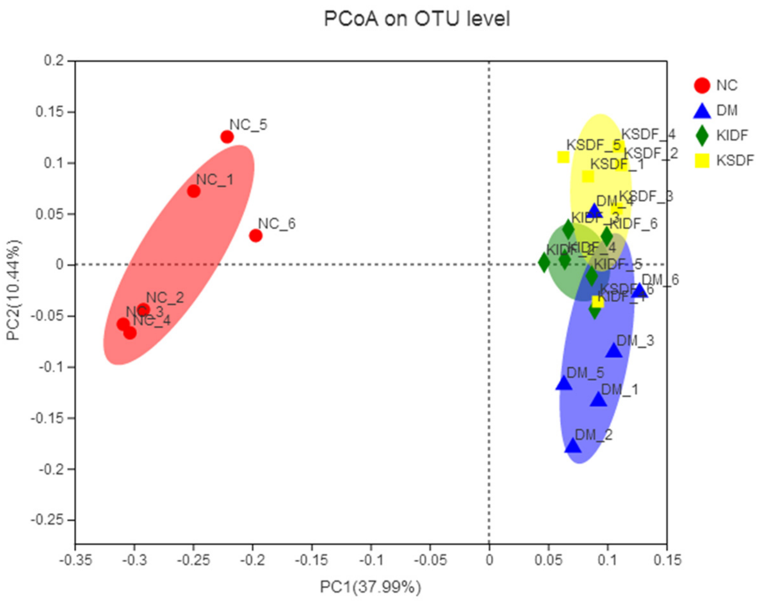

Figure S3. Effects on the gut microbiota structure by beta diversity metric using unsupervised multivariate statistical methods with principal coordinate analysis (PCoA) among the normal control group (NC, n = 6), diabetes model group (DM, n = 6), kiwifruit insoluble dietary fiber treatment group (KIDF, n=6) and kiwifruit soluble dietary fiber treatment group (KSDF, n = 6).

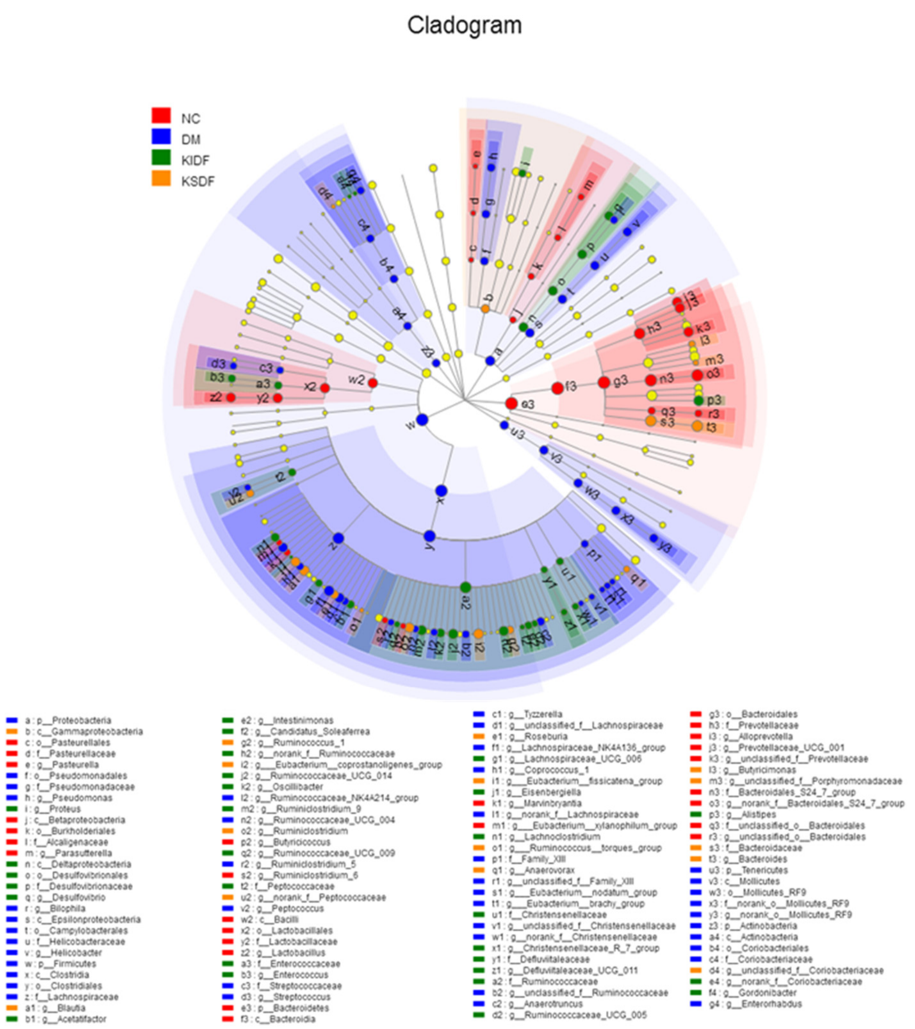

Figure S4. The significantly different species were determined based on the LEfSe method using the nonparametric factorial Kruskal-Wallis rank sum test at a significance level of 0.05. LEfSe taxonomy cladogram: different colors suggest enrichment of certain taxa in control group (NC, green, n = 6), metformin (MET, red, n = 6), and pumpkin polysaccharide (PPS; blue, n = 6). The size of the circles is based on relative abundance.

Table S1. Monosaccharide composition of KSDF and KIDF.

%	KSDF	KIDF
Rhamnose	3.76±0.13	3.01±0.23
Fucose	17.11±0.78	9.82±0.15
Arabinose	5.23±0.72	9.31±0.39
Xylose	16.11±0.47	36.35±0.96
Mannose	4.76±0.16	3.09±0.14
Glucose	4.73±0.46	24.59±0.3
Galactose	24.49±0.22	6.59±0.41
Galacturonic acid	23.81±0.28	7.24±0.28

All data are presented in the manner of means ± SD (n = 3). KSDF, kiwifruit soluble dietary fiber; KIDF, kiwifruit insoluble dietary fiber.

Table S2. Ingredients and energy densities of experimental diets.

Ingredients	D12450J		D12492	
	g%	kcal%	g%	kcal%
Casein	0.190	0.198	0.258	0.198
L-cystine	0.003	0.003	0.004	0.003
Sucrose	0.336	0.351	0.094	0.072
Corn starch	0.299	0.312	-	-
Lodex 10 sugar	0.033	0.035	0.162	0.124
Cellulose	0.047	0.000	0.065	0.000
Soybean oil	0.024	0.056	0.032	0.056
Lard	0.019	0.045	0.317	0.547
Mineral mixture	0.047	0.000	0.065	0.000
Hydrocholine tartrate	0.002	0.000	0.003	0.000
Vitamin mixture	0.001	0.000	0.001	0.000
Dye	0.000	0.000	0.000	0.000
Energy（kcal/g diet）	3.82		5.21	

Table S3. Component of the PCR reaction system.

Component	Volume
5×FastPfu buffer	4 μL
2.5 mM dNTPs	2 μL
forward primer（5 μM）	0.8 μL
reverse primer（5 μM）	0.8 μL
BSA	0.2 μL
FastPfu polymerase	0.4 μL
DNA template	10 ng
Total volume	20 μL (With ddH2O complement)

Table S4. Correlation of key communities of gut microbiota and biochemical parameters in NC, DM, KIDF and KSDF groups.

Taxon	Spearman' correlation R													
	Glu	HOMA-IR	TNF-α	IL-6	TG	TC	LDL	Acetic acid	Propionic acid	Butyric acid	Isobutyric acid	Isovaleric acid	Valeric acid	Total SCFAs
Enterorhabdus	0.54**	0.43*	0.55**	0.68***	0.48*	0.55**	0.65***	-0.28	-0.13	-0.38	-0.35	-0.48*	-0.22	-0.4
Bacteroides	0.52**	0.57**	0.42*	0.45*	0.28	0.39	0.42*	-0.6**	-0.01	-0.04	-0.63***	-0.42*	-0.12	-0.32
Butyricimonas	0.04	0.07	0.17	0.07	0.13	-0.01	0.15	-0.54**	-0.18	0.2	-0.46*	-0.52**	-0.21	-0.26
Alloprevotella	-0.66***	-0.68***	-0.65***	-0.62**	-0.51*	-0.61**	-0.51*	0.47*	-0.07	0.52**	0.39	0.33	-0.06	0.44*
Prevotellaceae_UCG-001	-0.57**	-0.54**	-0.57**	-0.55**	-0.62**	-0.54**	-0.59**	0.45*	0.18	0.24	0.56**	0.35	-0.12	0.42*
Alistipes	-0.51*	-0.50*	-0.54**	-0.66***	-0.36	-0.44*	-0.39	0.09	0	0.5*	0.38	-0.16	0.27	0.25
Enterococcus	0.39	0.31	0.50*	0.38	0.47*	0.45*	0.36	-0.22	-0.08	-0.39	-0.24	-0.59**	0.2	-0.39
Lactobacillus	-0.35	-0.28	-0.16	-0.16	-0.25	-0.16	-0.39	0.59**	0.35	-0.02	0.23	0.58**	0.16	0.49*
Streptococcus	0.64***	0.64***	0.70***	0.68***	0.63***	0.59**	0.54**	-0.45*	0.04	-0.52**	-0.49*	-0.58**	-0.13	-0.47*
Christensenellaceae_R-7_group	0.46*	0.46*	0.20	0.34	0.18	0.37	0.41*	-0.47*	-0.31	0.05	-0.33	-0.43*	-0.19	-0.35
Defluviitaleaceae_UCG-011	0.48*	0.39	0.36	0.42*	0.32	0.42*	0.58**	-0.47*	-0.26	-0.21	-0.24	-0.63***	-0.13	-0.48*
Eubacterium_xylanophilum_group	-0.32	-0.29	-0.36	-0.36	-0.47*	-0.39	-0.45*	0.25	0.07	0.23	0.38	0.47*	0.16	0.32
Blautia	0.41*	0.37	0.40	0.43*	0.38	0.62**	0.39	0.02	0.07	-0.09	-0.45*	-0.51*	0.34	-0.06
Coprococcus_1	0.49*	0.35	0.54**	0.52**	0.37	0.36	0.53**	-0.26	-0.22	-0.22	-0.19	-0.41*	-0.2	-0.39
Lachnoclostridium	0.53**	0.58**	0.59**	0.59**	0.62**	0.67***	0.55**	-0.51*	-0.31	-0.2	-0.47*	-0.62**	0.24	-0.47*
Lachnospiraceae_NK4A136_group	0.24	0.39	0.67***	0.43*	0.44*	0.46*	0.25	-0.3	-0.02	-0.33	-0.48*	-0.08	0.18	-0.3
Marvinbryantia	-0.40	-0.30	-0.16	-0.25	-0.15	-0.14	-0.38	0.3	-0.13	-0.15	0.47*	0.5*	0.03	0.03
Roseburia	0.53**	0.59**	0.34	0.39	0.35	0.37	0.37	-0.62**	-0.12	0.16	-0.54**	-0.59**	0.11	-0.23
Tyzzerella	0.51*	0.6**	0.59**	0.53**	0.65***	0.69***	0.39	-0.43*	-0.29	-0.14	-0.77***	-0.45*	0.23	-0.37
Peptococcus	0.53**	0.54**	0.72***	0.62**	0.52**	0.71***	0.55**	-0.42*	-0.14	-0.44*	-0.58**	-0.51*	0.22	-0.5*
Eubacterium_coprostanoligenes_group	-0.10	-0.13	-0.32	-0.24	-0.41*	-0.21	-0.08	0.05	0.22	0.53**	-0.08	-0.18	-0.03	0.35
Butyricicoccus	-0.40	-0.38	-0.41*	-0.46*	-0.35	-0.35	-0.43*	0.65***	0.29	0.23	0.35	0.58**	0.29	0.53**
Intestinimonas	0.6**	0.58**	0.55**	0.53**	0.57**	0.61**	0.45*	-0.5*	-0.24	-0.1	-0.48*	-0.75***	0.19	-0.39
Oscillibacter	0.57**	0.61**	0.48*	0.49*	0.51*	0.54**	0.48*	-0.57**	-0.15	-0.05	-0.62**	-0.69***	0.24	-0.34
Ruminiclostridium_6	-0.19	-0.12	-0.16	-0.20	-0.19	0.01	-0.25	0.19	-0.14	0.5*	-0.2	-0.06	0.22	0.26

Ruminococcus_1	-0.41*	-0.46*	-0.41*	-0.43*	-0.42*	‘-0.50*	-0.49*	0.37	0	0.49*	0.16	0.1	-0.09	0.4
Parasutterella	-0.59**	-0.66***	-0.58**	-0.56**	-0.59**	-0.73***	-0.48*	0.43*	0.05	0.28	0.65***	0.57**	-0.21	0.38
Bilophila	0.72***	0.75***	0.61**	0.74***	0.70***	0.55**	0.59**	-0.48*	-0.19	-0.41*	-0.53**	-0.23	-0.21	-0.51*
Desulfovibrio	0.42*	0.37	0.46*	0.35	0.35	0.43*	0.41*	-0.35	-0.07	-0.23	-0.3	-0.68***	0.06	-0.37
Helicobacter	0.54**	0.59**	0.52**	0.50*	0.52**	0.51*	0.41*	-0.3	-0.09	-0.17	-0.58**	-0.27	0.16	-0.32

*Spearman’s correlation is significantly different at a level of 0.05; ** Spearman’s correlation is significantly different at a level of 0.01; *** Spearman’s correlation is significantly different at a level of 0.001. For the limited table, only the significant Spearman’s correlation R values were showed.

Table S5. Correlation of SCFAs and biochemical parameters in NC, DM, KIDF and KSDF groups.

name	Glu	HOMA-IR	TNF-α	IL-6	TG	TC	LDL
Acetic_acid	-0.59**	-0.69***	-0.53**	-0.55**	-0.57**	-0.43*	-0.58**
Propionic_acid	-0.26	-0.27	-0.23	-0.22	-0.47*	-0.31	-0.22
Butyric_acid	-0.51*	-0.48*	-0.68***	-0.69***	-0.64***	-0.6**	-0.59**
Isobutyric_acid	-0.46*	-0.62**	-0.63**	-0.57**	-0.57**	-0.56**	-0.45*
Isovaleric_acid	-0.42*	-0.39	-0.34	-0.33	-0.32	-0.39	-0.41*
Valeric_acid	-0.2	-0.08	-0.06	-0.26	-0.05	0.15	-0.25
Total_SCFAs	-0.65***	-0.65***	-0.7***	-0.7***	-0.82***	-0.65***	-0.68***

*Spearman's correlation is significantly different at a level of 0.05; ** Spearman's correlation is significantly different at a level of 0.01; *** Spearman's correlation is significantly different at a level of 0.001. For the limited table, only the significant Spearman's correlation R values were showed.
